# A single-center analysis of genotype–phenotype characteristics of Chinese patients with autosomal dominant polycystic kidney disease by targeted exome sequencing

**DOI:** 10.3389/fgene.2022.934463

**Published:** 2022-09-15

**Authors:** Ziyan Yan, Yuchen Wang, Wenfeng Deng, Yi Zhou, Yangcheng Hu, Ka Qi, Ding Liu, Renfei Xia, Rumin Liu, Wenli Zeng, Wei Zhang, Jian Xu, Fu Xiong, Yun Miao

**Affiliations:** ^1^ Department of Transplantation, Nanfang Hospital, Southern Medical Univerisity, Guangzhou, China; ^2^ Hemodialysis Center, Qinhuangdao Charity Hospital, Qinhuangdao, China; ^3^ Division of Transplantation, Zhujiang Hospital, Southern Medical University, Guangzhou, China; ^4^ Guangzhou Jiajian Medical Testing Co Ltd, Guangzhou, China; ^5^ Department of Medical Genetics, School of Basic Medical Sciences, Southern Medical University, Guangzhou, China

**Keywords:** autosomal dominant polycystic kidney disease, PKD1, PKD2, mutation, TES using NGS

## Abstract

**Background:** Autosomal dominant polycystic kidney disease (ADPKD) is mainly caused by *PKD1* and *PKD2* mutations. However, only a few studies have investigated the genotype and phenotype characteristics of Asian patients with ADPKD. This study aimed to investigate the relationship between the natural course of ADPKD genotype and phenotype.

**Methods:** Genetic studies of *PKD1/2* genes of Chinese patients with ADPKD in a single center were performed using targeted exome sequencing and next-generation sequencing on peripheral blood DNA.

**Results:** Among the 140 patients analyzed, 80.00% (*n* = 112) harbored *PKD1* mutations, 11.43% (*n* = 16) harbored *PKD2* mutations, and 8.57% (*n* = 12) harbored neither *PKD1* nor *PKD2* mutations. The average age at dialysis was 52.60 ± 11.36, 60.67 ± 5.64, and 52.11 ± 14.63 years, respectively. The renal survival rate of ADPKD patients with *PKD1* mutations (77/112) was significantly lower than that of those with *PKD2* mutations (9/16), leading to an earlier onset of end-stage renal disease (ESRD). Renal prognosis was poor for those with nonsense mutations, and they required earlier renal replacement therapy.

**Conclusions:** The genotype and phenotype characteristics of ADPKD patients potentially vary across ethnic groups. Our findings supplement the genetic profiles of Chinese ADPKD patients, could serve as a guide for therapy monitoring and prognosis assessment of ADPKD, and may improve the clinical diagnosis.

## Introduction

Autosomal dominant polycystic kidney disease (ADPKD) is a common genetic renal disease, with an incidence of one per 1000 to 2500 individuals. It is characterized by progressive renal cyst enlargement, and approximately 50% of patients progress to end-stage renal disease (ESRD) by age 60 (Bergmann, 2015; Cornec-Le et al., 2019). ADPKD is a systemic disease that may cause various extrarenal complications, including hypertension, intracranial aneurysm, and heart valve disease (Lanktree and Chapman, 2017; Cornec-Le et al., 2019; Li et al., 2021).

ADPKD is mainly caused by mutations in *PKD1* (in approximately 80% of disease pedigrees) or *PKD2* (in approximately 15% of disease pedigrees). In the remaining 5–10%, rare or unknown mutations (e.g., *GANAB* and *HNF1B*) are involved (Besse et al., 2017; Cornec-Le et al., 2019). *PKD1* is located on chromosome 16 (16p13.3) and encodes polycystin-1 (PC1), the 11th transmembrane protein. In contrast, *PKD2* is located on chromosome 4 (4q21) and encodes polycystin-2 (PC2), a member of the transient receptor potential family of non-selective ion channels (Harris and Torres, 2009). Both PC1 and PC2 are located on the renal primary cilia, which is a dose-dependent mechano-sensor that regulates the differentiation and proliferation of renal tubular epithelial cells. Cystogenesis occurs when the concentration of PC1 or PC2 decreases below a certain threshold (Audrézet et al., 2016).

Previous studies have demonstrated the association of renal function with various genotypes and some mutation types. For example, patients with *PKD2* mutations reached ESRD 20 years later than those with *PKD1* mutations, and those with *PKD1* non-truncating (*PKD1*-NT) mutations progressed to ESRD 12 years later than those with truncating mutations (Cornec-Le et al., 2019). These findings indicate the significance of ADPKD genotypes in predicting renal prognosis.

However, thus far, the available studies on *PKD1*/*PKD2* mutations and clinical phenotypes have been mainly conducted in Western countries, and only a few studies have investigated the genotype and phenotype characteristics of Asian patients with ADPKD. Therefore, this study aimed to examine Chinese patients with ADPKD at a single center to explore the relationship between the natural course of ADPKD and genotype and phenotype. Our findings contribute new information on the genetic profiles of Chinese ADPKD populations.

## Materials and methods

### Patients

We retrospectively examined 140 ADPKD patients in the Nanfang Hospital (Guangzhou, China) between November 2013 and July 2018. ADPKD was diagnosed using the following criteria: presence of at least two renal cysts (unilateral or bilateral) in individuals aged <30 years; presence of at least two cysts in each kidney among those aged 30–59 years; and at least four cysts in each kidney among those aged ≥60 years (Ravine et al., 1994). Peripheral blood was collected from each patient, and baseline demographic characteristics (age and sex), age at ESRD, initiation of renal replacement therapy (RRT), and family history of ESRD/ADPKD were recorded.

### Target gene enrichment, library construction, and capture sequencing

DNA was extracted from peripheral blood samples using a Solpure Blood DNA kit (Magen, Wuhan, China). The amplified DNA band was interrupted into small DNA fragments with a main band of less than 500 bp and a peak value of 350 bp for library preparation. Briefly, fragments were blunted, and an “A” was added to the 3′ end to connect to a special linker with a “T” base at the 5′ end. The library with adapters was amplified using pre-capture ligation-mediated PCR (LM-PCR). The fragment size of the library was determined by agarose gel electrophoresis, and the concentration of the library was determined by Qubit3.0 and real-time PCR. The library that passed the quality inspection was then diluted to the required concentration for testing, and sequencing analysis was carried out using the Illumina NextSeq 500 sequencing platform. Sequencing results were analyzed using bioinformatics and interpreted by a genetic counselor.

### Analysis of next-generation sequencing (NGS) results

The annotation scope of the raw data included the variation in each exon and the variation in the 10 bp upstream and downstream introns of the exon. Variation types included frameshift, splicing, nonsense, missense, atypical, synonymous, and in-frame insertions/detection. Quality control was conducted on the variant data. The ratio of the sequencing depth of each exon of the PKD1/2 gene is fixed for each sample (if there is no large fragment deletion or duplication). When the sequencing depth ratio of several consecutive exons (usually three or more) between PKD1 and PKD2 genes deviates from the baseline value, a large deletion will be considered, and further confirmation will be conducted by qRT-PCR accordingly. If not, we then compared the hg19 version human genome reference sequence of *PKD1* and *PKD2* gene sequences; searched the internal database (Guangzhou Jiajian medicine), dbSNP, esp6500, exac, and other population databases; and determined the insertion or deletion of small fragments (indel variation) and mononucleotide polymorphism. Variations with a minor allele frequency of <1% in gnomAD (low-frequency benign variation), artifacts, and variations in highly homologous regions were removed. Subsequently, SIFT and Polyphen2 were used to predict the conservation/pathogenicity/harmfulness of variants. The HGMD, PubMed, ClinVar, and other databases, as well as variant-related literature, were also searched. Variant classification was conducted according to the American Society for Medical Genetics and Genomics (ACMG) guidelines and reported as pathogenic, likely pathogenic, variant of unknown significance (VUS), likely benign, or benign.

### Statistical analysis

Categorical variables are reported as numbers (percentages), whereas continuous variables are reported as median (range) or mean ± SD. Renal prognosis was evaluated using Kaplan–Meier survival analysis and single-variate analysis using the Cox proportional hazard model. Statistical analysis was performed using SPSS 26.0 software, and *p*-values < 0.05 were considered statistically significant.

## Results

### Demographic characteristics

Among the 140 patients, 79 (56.43%) were men and 61 (43.57%) were women. The average age of men at the visit was 57.22 ± 13.47 years, while that of women was 57.44 ± 11.94 years. A total of 90 (64.29%) patients reported a family history of ADPKD, comprising 36 patients (40.00%, 36/90) with single-parent history only, 21 (23.33%, 21/90) with sibling history only, and 33 with both parent and sibling history (36.67%, 33/90) (Table 1).

**TABLE 1:** Cohort information on ADPKD patients included in this study (n = 140).

Group	Proportion	Average age	
*Demographic information, age (mean ± SD)*		
Male	79/140 (56.43%)	57.22 ± 13.47
Female	61/140 (43.57%)	57.44 ± 11.94
*RRT distribution in different genotype, RRT age* (*mean ± SD*)			
*PKD1*	77/112 (68.75%)	52.60 ± 11.36
*PKD2*	9/16 (56.25%)	60.67 ± 5.64
Others	9/12 (75.00%)	52.11 ± 14.63

a ADPKD: autosomal dominant polycystic kidney disease, RRT: renal replacement therapy.

### Genotype distribution

**FIGURE 1:**
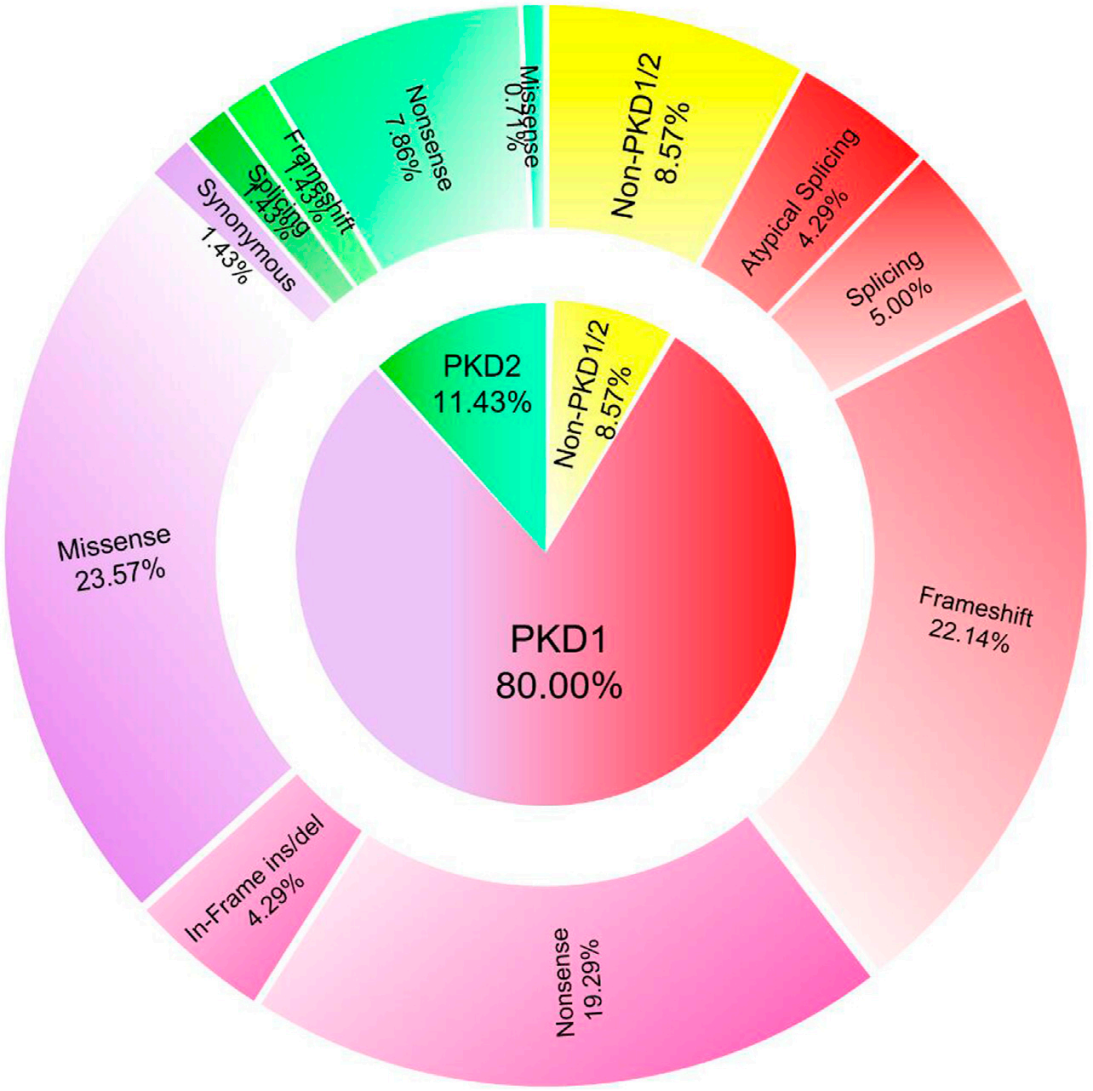
Distribution of genotypes and mutation types in ADPKD patients included in this study.

Genetic analysis showed that 80.00% (*n* = 112) of the included patients harbored *PKD1* mutations and 11.43% (*n* = 16) harbored *PKD2* mutations, whereas no mutation was detected in the remaining 8.57% (*n* = 12). Among the 112 patients with *PKD1* mutations, 58.04% (*n* = 65) had truncating mutations (*PKD1*-T). In contrast, among the 16 patients with *PKD2* mutations, 93.75% (*n* = 15) had truncating mutations (*PKD2*-T). Furthermore, *PKD1*-missense, *PKD1*-frameshift, and *PKD1*-nonsense mutations were the most common mutations detected in this study, accounting for 25.78% (33/128), 24.22% (31/128), and 21.09% (27/128), respectively (Figure 1). The genotypes and mutation types of the study population are presented in Supplementary Table S1.

### *PKD1/PKD2* genotypes as renal prognostic indicators in ADPKD patients

As of July 2018, 67.86% (95/140) of patients progressed to ESRD and required RRT (i.e., hemodialysis, peritoneal dialysis, hemofiltration, hemodiafiltration, and kidney transplantation), including 77 patients with *PKD1* mutations, nine with *PKD2* mutations, and nine without *PKD1*/*PKD2* mutations. The average age of dialysis patients was 52.60 ± 11.36, 60.67 ± 5.64, and 52.11 ± 14.63 years, respectively. Among them, 61.05% (58/95) were men and 38.95% (37/95) were women, with an average age of 54.78 ± 12.05 and 51.03 ± 10.34 years, respectively.

Of the 77 patients with *PKD1* mutations, the average age of RRT initiation in 46 patients (59.74%) with *PKD1*-T mutations was 52.13 ± 8.29 years, while that in 31 patients (40.26%) with *PKD1*-NT mutations was 53.29 ± 14.76 years.

**FIGURE 2:**
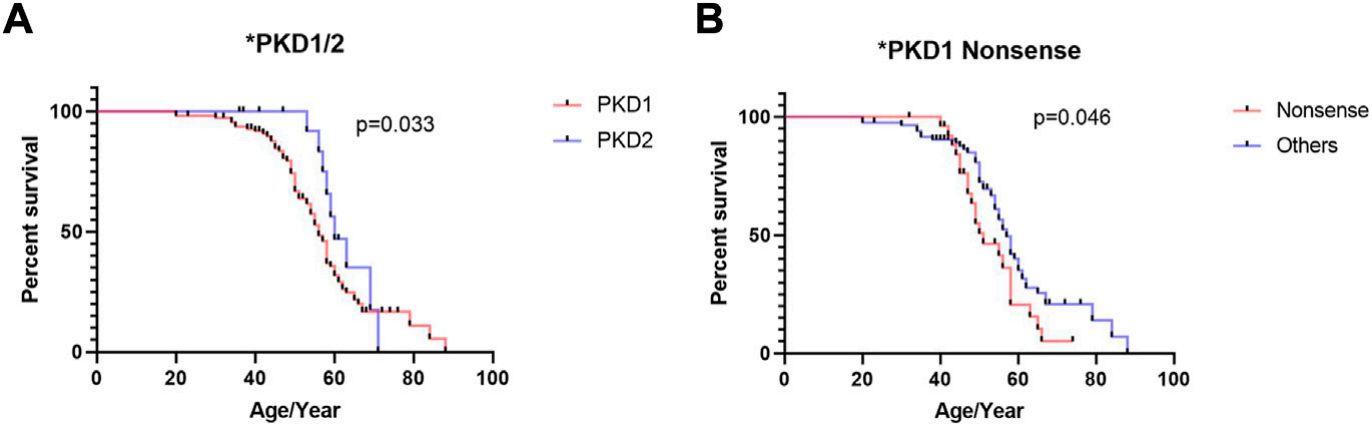
Kaplan–Meier survival analysis of ADPKD patients stratified by genotypes and mutation types. **(A)** Comparison of patients with PKD1 gene mutation and PKD2 gene mutation **(B)** Comparison of patients with PKD1 gene nonsense mutation and PKD1 gene other mutation. The end-point event was the start of regular renal replacement therapy.

The results of univariate Cox regression analysis in patients with *PKD1* are shown in Table 2. Kaplan–Meier survival analysis revealed that renal survival is significantly worse in patients with *PKD1* mutations than in those with *PKD2* mutations, and patients with *PKD1* mutations reached ESRD earlier (Figure 2A, *p* = 0.030). In the *PKD1* cohort, the renal survival rate was significantly lower in patients with nonsense mutations than in those with other mutation types, thus requiring RRT earlier (Figure 2B, *p* = 0.046). However, other mutation types had no marked effect on renal prognosis. Meanwhile, sex or mutation position did not significantly affect the prognosis of ADPKD patients.

**TABLE 2:** Univariate analysis of risk factors related with RRT in the *PKD1* cohort.

Variable	Univariate analysis	
	Hazard ratio (95% CI)	*p*
Truncating	1.438 (0.896–2.308)	0.132
Mutation type		
Nonsense	1.646 (0.990–2.737)	0.055
Frameshift	1.062 (0.635–1.776)	0.820
Missense	0.704 (0.421–1.179)	0.182
In-frame insertions/deletions	0.688 (0.216–2.192)	0.527
Splicing	0.823 (0.332–2.043)	0.675
Synonymous	2.041 (0.498–8.374)	0.322
Atypical Splicing	0.795 (0.250–2.530)	0.698
Mutation Position		
*PKD1* N-terminus domain	1.341 (0.808–2.225)	0.257
PKD domain	0.735 (0.337–1.603)	0.439
REJ domain	0.776 (0.426–1.414)	0.408
TM domain	1.126 (0.707–1.794)	0.617
*PKD1* C-terminus domain	0.727 (0.228–2.315)	0.589

a RRT: renal replacement therapy, CI: confidence interval.

### ACMG variants as renal prognostic indicators in ADPKD patients

**FIGURE 3:**
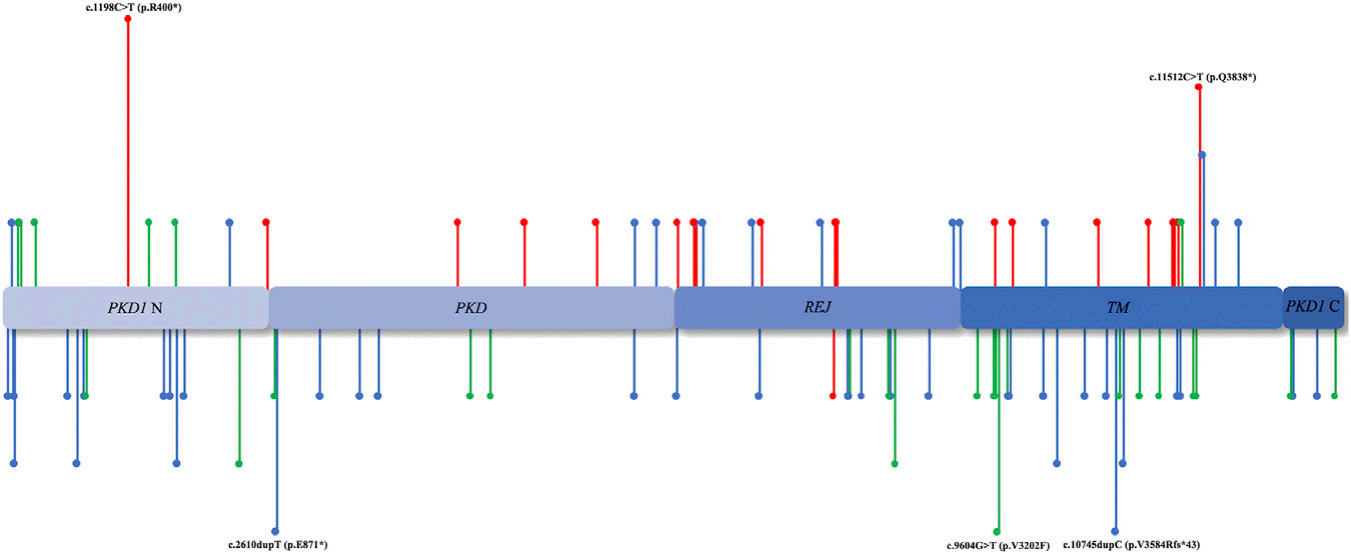
Mutation region. The distribution of *PKD1/PKD2* mutation sites in various domains. The unreported mutation sites are annotated below, whereas those reported previously are annotated above. The red lines represent ACMG I, the blue lines represent ACMG II, and the green lines represent ACMG III.

According to the ACMG classification (Hampel et al., 2015), of the 128 mutations identified in the *PKD1/PKD2* cohort, 28.12% (*n* = 36) were pathogenic, 48.44% (*n* = 62) were likely pathogenic, and 23.44% (*n* = 30) were VUS (Figure 3). Among the 112 patients with *PKD1*, the median age of ESRD onset in those with pathogenic, likely pathogenic, and VUS mutations was 49.5 (20–67), 54 (20–84), and 56 (30–88) years, respectively. In the 16 patients with *PKD2*, only “pathogenic” (*n* = 11) and “likely pathogenic” mutations (*n* = 5) were detected.

## Discussion

Stratifying the ADPKD population is a prerequisite for personalization of clinical therapy and patient care. Therefore, understanding the characteristics and clinical course of ADPKD patients is critical for selecting appropriate clinical interventions. The available genetic profiles of ADPKD patients available in the literature are mainly applicable to Western populations, and only a few studies have reported the genotype and phenotype characteristics of Asian patients (Sha et al., 2017). To uncover the genetic profiles of Asian ADPKD populations, we characterized the genotype, mutation type, and mutation position based on targeted exome sequencing (TES) using NGS data and analyzed the prognostic differences in renal function among Chinese ADPKD populations.

The large size, complexity, and high GC content of *PKD1* increase the difficulty of mutation detection (Harris and Torres, 2009). *PKD1* contains 46 exons, and the duplication of its first 33 exons with six pseudogenes, which shares ∼98% DNA sequence identity in the homology region, easily causes false-positive and false-negative results in genotype detection (Bogdanova et al., 2001; Song et al., 2017). In addition, as of October 2021, 1273 *PKD1*-related pathogenic mutations and 202 *PKD2*-related pathogenic mutations were recorded in the ADPKD database (PKDB, http://pkdb.mayo.edu). The high allelic heterogeneity between *PKD1* and *PKD2* creates challenges in the molecular diagnosis of ADPKD. Thus, a faster, more sensitive, and more economical assay for diagnosis and mutation screening of ADPKD is urgently required.

TES using NGS data is regarded as an effective method for investigating monogenic diseases. The coding regions of all target genes were first amplified by an LR-PCR-based method and then sequenced by an NGS platform, which can effectively avoid the interference of homologous sequences. In contrast, both whole-genome sequencing (WGS) and whole-exome sequencing (WES) sequencing failed to effectively avoid the interference of homologous sequences, and the cost of a single sequencing was higher. Due to the long sequence of the coding region of the target gene, one-generation sequencing is expensive and high throughput cannot be achieved. Therefore, with only a few genes and exons related to the disease being considered in TES (Ali et al., 2019), it allows automatic analysis of large amounts of data in a short period of time, as well as the mutation screening of various cystic diseases and potential modification genes. In this way, this method may revolutionize the genetic testing technology for ADPKD owing to its high accuracy and low cost.

Furthermore, 91.43% (128/140) of the mutations were detected in *PKD1*/*PKD2*, including 72 (56.25%) unreported mutations. We explain the 8.57% (12/140) cases of no mutations detected as follows. First, mutations in rare genes may be responsible for cystic diseases rather than *PKD1*/*PKD2* (i.e., *ALG8*, *ALG9*, *GANAB*, *PRKCSH*, *SEC6A*, and *SEC63*) (Besse et al., 2017; Besse et al., 2019). Second, somatic mosaicism, specifically variable involvement of affected cells, caused a low signal-to-noise ratio, thus making it difficult to detect mutations (Iliuta et al., 2017). Last, there might be omission of some deep intronic or pathogenic mutations in the regulatory region of *PKD1*/*PKD2*.

Currently, several international studies on genotype and phenotype characteristics have demonstrated the strong correlation of genotype and mutation type with the indicators used to assess the severity of renal diseases, including estimated glomerular filtration rate, height-adjusted total kidney volume, and the age of ESRD onset. For example, renal prognosis was worse in patients with *PKD1* than in those with *PKD2*, with a median age of ESRD onset of 58 and 79 years, respectively (Cornec-Le et al., 2019). Furthermore, the median age of patients with truncating mutations (i.e., splicing, frameshift, and nonsense) and non-truncating mutations (i.e., missense, in-frame insertions/detection, and atypical splicing) in *PKD1* was 55.6 and 67.9 years, respectively (Cornec-Le et al., 2019).

However, consistent with those of previous studies on the Asian ADPKD population (Dechao et al., 2018), our results also showed that the Chinese ADPKD study population reached ESRD earlier than international ADPKD populations. In this study, the median ages of *PKD1* and *PKD2* patients reaching ESRD were 54 and 58 years, respectively. Additionally, the renal survival rate of patients with *PKD1* nonsense mutations was significantly lower than that of patients with other *PKD1* mutations, whereas that of patients with other mutation types showed no significant difference. For example, we did not find a significant correlation between the occurrence of truncation mutations and renal prognosis in ADPKD patients, which was inconsistent with previous studies in the Asian population (Dechao et al., 2018). Hence, the genotype and phenotype characteristics of ADPKD patients vary across ethnic groups.

Moreover, modification effects have been demonstrated in ADPKD patients with the same mutation as the main effects (Cornec-Le et al., 2018). The ACMG has developed guidelines for explaining rare sequence variants, which are classified as “pathogenic,” “likely pathogenic,” “VUS,” “likely benign,” and “benign.” However, some variants have been challenged because of the high cumulative incidence of “likely pathogenic” variants in *PKD1*/*PKD2*.

In this study, among the patients with *PKD1* mutations requiring RRT, the proportion of those with “pathogenic,” “likely pathogenic,” and “VUS” variants was 72.00% (18/25), 68.42% (39/57), and 66.67% (20/30), respectively, with corresponding median ages of ESRD onset of 49.5, 54, and 56 years, respectively. Despite their conformity with the classification of ACMG, the incidence of ADPKD and the proportion of RRT in those with “likely pathogenic” and “VUS” variants both exceeded the estimates reported by epidemiological studies (Cornec-Le et al., 2018). In addition, among the patients with *PKD2* mutations, the proportion of those with “pathogenic” and “likely pathogenic” variants requiring RRT was 45.45% (5/11) and 80.0% (4/5), respectively. Therefore, renal prognosis assessment of *PKD1*/*PKD2* mutation carriers or ADPKD patients based on the ACMG variant classification needs to be reconsidered.

Our findings indicate that the mutation position of *PKD1*/*PKD2* has no significant association with disease progression, which is consistent with the results of previous retrospective studies (Cornec-Le et al., 2013; Ali et al., 2019). According to the two-hit hypothesis, germline mutations inactivate the *PKD1* allele, regardless of the mutation position. Subsequently, a cyst developed in the absence of PC1 with a second inactivating mutation. In contrast, some hold opposing perspectives: the mutation location significantly influences the renal phenotype. Rossetti et al. suggested that the mutant gene might generate a functional product, explaining why patients with a 5′ mutant progressed to a more severe phenotype than those with a 3′ mutant (Hateboer et al., 2000; Rossetti et al., 2002).

In conclusion, we conducted a preliminary study on the genotype and phenotype characteristics of Chinese ADPKD patients based on TES using NGS data. Our findings provide new insights into the genetic profiles of Asian ADPKD populations. Our findings strongly suggest that TES using NGS data could be the main method of ADPKD genetic investigation owing to its high precision, economic feasibility, and possibility of simultaneous mutation screening for various cystic diseases and potential modification genes. Hence, this method would contribute to the clinical diagnosis, treatment, and assessment of renal prognosis of ADPKD patients.

## Data Availability

The PKD1/2 genes-targeted exome sequencing data of patients are available at NCBI Sequence Read Archive with the BioProject accession number of PRJNA875618.
